# Reporting of MMR evidence in professional publications: 1988–2007

**DOI:** 10.1136/adc.2008.154310

**Published:** 2009-05-03

**Authors:** S Hilton, K Hunt, M Langan, V Hamilton, M Petticrew

**Affiliations:** 1 MRC Social and Public Health Sciences Unit, Glasgow, UK; 2 VRH Information Services, University of Glasgow, Glasgow, UK; 3 London School of Hygiene and Tropical Medicine, London, UK

## Abstract

**Objective:**

To examine how journals and magazines disseminate research evidence and guidance on best practice to health professionals by aligning commentaries on measles, mumps, and rubella vaccine (MMR) evidence in journals with key events in the MMR controversy.

**Design:**

Content analysis.

**Data sources:**

Comment articles in six commonly read UK publications.

**Main outcome measures:**

Number of comment pieces by publication, year and article type; trends in the focus, tone and inclusion of recommendations on MMR.

**Results:**

860 articles met the inclusion criteria (*BMJ* n = 104, *Community Practitioner* n = 45, *Health Visitor* n = 24, *Practice Nurse* n = 61, *Nursing Standard* n = 61 and *Pulse* n = 565). Of these 860 comment pieces, 264 made some reference to evidence endorsing the safety of MMR. Around one in 10 were rated as negative (10.9%, n = 29) or neutral (11.3%, n = 30) in relation to MMR safety, and nearly a quarter (22.7%, n = 60) were rated as mixed. Following the publication of Wakefield *et al*’s 1998 paper there was a period of neutrality. In 2000, despite growing public concerns and widespread media coverage, fewer than 20 comment pieces were published. Less than a quarter of comment pieces (n = 196, 22.7%) included recommendations.

**Conclusion:**

While a period of neutrality may reflect a professional response to uncertainty by holding back until consensus emerges, it may also represent a missed opportunity to promote evidence-based practice.

Clinical journals are an important vehicle for the dissemination of research findings to health professionals. However there is debate about how effective such outlets are in bridging the gap between evidence and practice,^1^ ^2^ ^3^ ^4^ and practice often lags behind the evidence. Evidence-based practice can be fostered by encouraging journals to disseminate information in a way that would motivate practitioners to change practice,^4^ including using editorials and commentary columns of journals and other publications for health professionals, particularly when there is uncertainty about the evidence base. Journals themselves identify supporting practitioners as their role; the *BMJ* identifies “Helping doctors make better decisions” as one of its objectives, and the magazine *Pulse* brands itself as “Informing, supporting, championing” GPs, while *Nursing Standard* states that it brings its readers “exclusive, up-to-the-minute coverage on issues affecting nursing practice”.

This study explores how these publications do this by examining the alignment between trends in their reporting of measles, mumps, and rubella vaccine (MMR) evidence, and important events in MMR vaccination “controversy” between 1988 and 2007 (see fig 1). This controversy was fuelled by the publication in 1998 of a paper raising the possibility of a link between the MMR vaccine, bowel disease and autism,^5^ contributing to a decline in MMR uptake, despite extensive evidence about MMR safety. Primary healthcare professionals have described a lack in confidence as they tried to advise parents on MMR safety during this period.^6^

**Figure 1:**
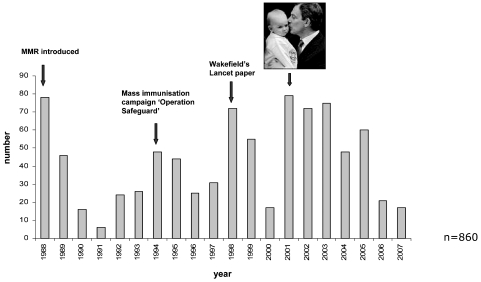
Comment pieces (n = 860) published between 1988 and 2007 on childhood immunisation in 60 publications.

## Methods

The most commonly-read journals and magazines aimed at community practitioners were identified through telephone interviews with health visitors, practice nurses, general practitioners and medical librarians (n = 20) working in Scotland and England, and through a survey (n = 185, response rate = 81.1%) conducted at the Community Practitioners’ and Health Visiting Association (CPHVA) 2007 annual conference. The six most commonly cited sources were: *BMJ* (sent weekly by the British Medical Association (BMA) to all BMA members and other subscribers), *Health Visitor*, *Community Practitioner* (previously *Health Visitor*, published fortnightly by the CPHVA), *Pulse* (a free weekly magazine sent to all GPs), *Practice Nurse* (published fortnightly) and *Nursing Standard* (published weekly). Each publication was reviewed to identify the sections that commented upon or distilled evidence. The journal sections identified for inclusion were editorials, commentaries, news, news analysis, reviews of clinical papers, education and debate articles, art and science articles and feature articles. Collectively we refer to these eligible articles as “comment pieces” We excluded letters, views, book reviews and primary research articles.

Initial searches showed that CINAHL and Embase offered the best coverage and indexing of these publications, so further searches in these databases were conducted from 1988 to 2007 to cover the whole period since the introduction of MMR into the childhood immunisation programme (CIP). (Full search strategy available from the authors.) Manual searches were also conducted. This process identified 936 potentially relevant commentaries.

Each comment piece was assessed by two reviewers for eligibility (n = 860). A coding frame (available on request) was developed, based on a random selection of 24 comment pieces from each journal, and tested and refined on subsequent samples to achieve consistency.

## Results

The majority of comment pieces were published in *Pulse* (65.7%, n = 565). The *BMJ* published 104 comment pieces (12.1%). *Practice Nurse* and *Nursing Standard* both published 61 pieces (7%), *Community Practitioner* published 45 (5.2%) and *Health Visitor* 24 (2.7) pieces. The most common article type was news articles (n = 657, 76.3%) followed by feature (n = 99, 11.5%), review (n = 40, 4.6%) commentary (n = 35, 4.0) and editorial (n = 29, 3.3%) pieces.

A time line detailing key events in relation to MMR is available from the authors. An initial peak in the number of commentaries in 1988 covering the introduction of MMR into the CIP was followed by an apparent reduction in interest until 1994, when the Department of Health introduced the school-based “MR catch-up”campaign, “Operation Safeguard”(fig 1). In 1998 there was another peak following the publication of Wakefield *et al*’s paper.^5^ In 2000, despite growing public concerns and widespread media coverage, fewer than 20 comment pieces were published, and in 2001 the debate became further politicised, illustrated by media debate about whether the British Prime Minister, Tony Blair, should reveal whether his baby son, Leo, had received the MMR vaccination. Media interest continued until 2005, declining thereafter.

### Trends in the main focus and tone of comment pieces

The most common focus of the 860 comment pieces was on the delivery of the CIP programme (n = 122; 14%). Most (n = 109; 12.6%) were published in *Pulse*. There were 75 comment pieces whose main focus was on endorsing MMR vaccination, while 50 (5.8%) primarily focused on potential adverse side effects.

Less than a quarter of comment pieces (n = 196, 22.7%) offered advice or recommendations. In the two-year period following the publication of the Wakefield paper in 1998 there were very few comment pieces containing recommendations, perhaps reflecting general uncertainty about MMR evidence among the editorial teams of these journals and magazines. Recommendations appeared more commonly during 2001 and 2002, although they still featured in only a minority of comment pieces.

### Trends in the tone of the comment pieces

**Figure 2:**
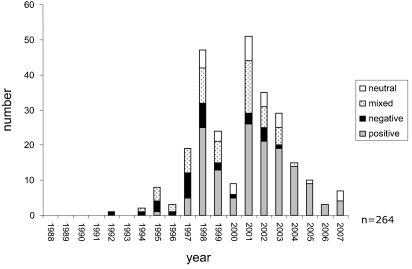
Trends in tone on MMR safety.

Of the 860 comment pieces, 264 (30.7%) made some reference to evidence endorsing the safety of MMR. Just over half of these 264 (54.9%, n = 145) were rated as having a positive tone. Around one in 10 were rated as negative (10.9%, n = 29) or neutral (11.3%, n = 30). Following the publication of Wakefield *et al*’s 1998 paper there was a period to 2004 during which many articles adopted a mixed, negative or neutral tone (fig 2). After this most of the comment pieces which made reference to the safety of MMR adopted a positive tone, and none was negative.

## Discussion

Our analysis highlights how uncertainty about evidence is reflected in the tone of professional journals and magazines. This was particularly evident in the period from 2001 to 2003 when many of the publications conveyed ambiguous messages about MMR safety, though there was little scientific disagreement that MMR was safe and there was scientific consensus from early on that the Wakefield study was neither generalisable nor robust. For at least two years following the publication of the Wakefield paper, there was also an information gap, with relatively few articles published.

There were surprisingly few journal editorials on MMR during this period, and only three appeared in nursing journals. This postponement of a robust defence of MMR may have contributed to undermining confidence among health professionals, particularly since health practitioners working at the “coal-face” needed immediate reassurances about MMR safety once the issue was raised in the popular press. While a period of neutrality may reflect a professional response to uncertainty by holding back until consensus emerges, it may also represent a missed opportunity to promote evidence-based practice. Indeed, over this period the main focus of articles in *Pulse* was on potential adverse side effects of MMR, a focus more closely aligned with the popular media’s representation that there were two competing bodies of MMR evidence,^7^ than with informing and supporting practitioners.

**What is already known on this topic**

- Health practitioners use the editorials and commentary sections of journals to help keep up to date with research developments and to inform their practice.
- These sources can play an important part in synthesising and disseminating guidance on current best practice to health practitioners, particularly when there is conflicting evidence, such as during a health controversy.

**What this paper adds**

- During the MMR controversy the slow response of journals and lack of recommendations may have added to clinical uncertainty, and increased the gap between evidence and practice.
- When new research attracts media attention but contradicts the existing evidence base, journals and magazines need to take a more proactive stance in translating the evidence to provide practitioners with clear guidance.

## Conclusions

Although the study is limited by the dominance in the data of one magazine (*Pulse*), the findings clearly highlight the tensions for journal editorial teams, and prompt reflection on how far journals need to go in leading opinion during public health controversies. The analysis also identifies clear missed opportunities to accurately inform practitioners about the evidence during this period, such as periods when some journals and magazines appeared to “stand back” and wait for consensus to develop. When controversy is at its height such uncertainty and neutrality may be perceived by public and practitioners as further evidence that a problem exists, and such voids may create new opportunities for alternative or speculative views to arise.
